# Feldenkrais Method Balance Classes Improve Balance in Older Adults: A Controlled Trial

**DOI:** 10.1093/ecam/nep055

**Published:** 2011-03-08

**Authors:** Karol A. Connors, Mary P. Galea, Catherine M. Said

**Affiliations:** ^1^Rehabilitation Sciences Research Centre, School of Physiotherapy, University of Melbourne, Parkville, VIC 3010, Australia; ^2^Calvary Health Care Bethlehem, Caulfield South, Australia; ^3^Austin Health, Heidelberg, Victoria, Australia

## Abstract

The objective of this study was to investigate the effects of Feldenkrais Method balance classes on balance and mobility in older adults. This was a prospective non-randomized controlled study with pre/post measures. The setting for this study was the general community. A convenience sample of 26 community-dwelling older adults (median age 75 years) attending Feldenkrais Method balance classes formed the Intervention group. Thirty-seven volunteers were recruited for the Control group (median age 76.5 years). A series of Feldenkrais Method balance classes (the 33312Getting Grounded Gracefully33313 series), two classes per week for 10 weeks, were conducted. Main outcome measures were Activities-Specific Balance Confidence (ABC) questionnaire, Four Square Step Test (FSST), self-selected gait speed (using GAITRite instrumented gait mat). At re-testing, the Intervention group showed significant improvement on all of the measures (ABC, *P* = .016, FSST, *P* = .001, gait speed, *P* < .001). The Control group improved significantly on one measure (FSST, *P* < .001). Compared to the Control group, the Intervention group made a significant improvement in their ABC score (*P* = .005), gait speed (*P* = .017) and FSST time (*P* = .022). These findings suggest that Feldenkrais Method balance classes may improve mobility and balance in older adults.

## 1. Introduction

Various forms of exercise have aimed to improve balance in older adults, generally to attempt to reduce the incidence of falls [1–4]. Exercise has also been proposed as a preventative strategy to slow the decline from pre-frailty to frailty in older adults [5]. Exercise approaches to achieve these aims have varied from strength and balance training [6] to specific balance exercises [3] to Tai Chi [7–9]. As yet, no single approach has emerged as being definitively more effective than another. However, a recent systematic review and meta analysis of studies using exercise to prevent falls, suggests that balance training may be more effective in lowering falls risk than other exercise components such as strength or endurance training [10].

The Feldenkrais Method has the potential to be a useful tool for balance retraining. The Feldenkrais Method was developed over several decades by Dr. Moshe Feldenkrais (1904–1984), an Israeli scientist and Judo master [11] with a pioneering interest in human movement from a dynamics systems perspective. Dr. Feldenkrais combined his understanding of human movement from his martial arts training, with extensive reading from Eastern and Western sources to develop a unique approach to improving movement. This approach is currently practised by thousands of registered Feldenkrais Method practitioners working in over 20 countries. The lessons are based on martial arts principles, but have been devised to address improvement in all aspects of human function, from an actor performing on stage to a disabled person turning over in bed [12]. The Feldenkrais Method `Awareness Through Movement' classes use an exploratory learning approach, in which participants are verbally guided through movement sequences aimed at improvement of body awareness and movement organization [13]. Stephens [14] has proposed that “this process facilitates the learning of strategies for improving organization and coordination of body movement by developing spatial and kinesthetic awareness of body-segment relationships” (page 1642). Among the hundreds of lessons which Dr. Feldenkrais created [12], many are suitable for balance retraining.

Recent investigations into the nature of balance have revealed its complexity [15]. Achieving effective balance is a multi-system and multi-dimensional task [16]. Not only are the motor, sensory (including proprioception, vision and vestibular systems) and cognitive systems of the body involved, but the ability to dynamically interact with the environment must also be included [15]. The Feldenkrais Method is an approach to balance retraining that is multi-dimensional. All parts of the body are potentially involved in the movements, including the eyes, the feet and the trunk, which are all important contributors to balance [17]. There is also involvement of the senses in the lessons, including tactile sensation, proprioception, vestibular stimulation and vision. A fundamental principle of the Feldenkrais Method is that the processes of thinking, feeling, sensing and doing are all interrelated components of human functioning, and to address any one component is to address them all [13]. It is this concept of the unity of the mind and body that distinguishes the Feldenkrais Method from most mainstream approaches to movement improvement.

There has been little high quality research into the Feldenkrais Method. A randomized controlled trial comparing the Feldenkrais Method to Tai Chi and a non-treatment control group, in a sample of 59 older women, found significant improvement in several measures of function and balance in the Feldenkrais Group and on one measure in the Tai Chi group and none in the control group [18]. However, statistical analysis did not extend to between group comparisons, so interpretation of the results is limited. The study also did not include any assessment of balance confidence, which is an important aspect of balance retraining. These results support the need for further studies into this approach to improving balance.

The most effective way to investigate the dynamic properties of balance and mobility, is to use dynamic balance tests [15]. The three primary outcome measures used in the current study were the Four Square Step test (FSST) [19], gait speed (measured using the GAITRite electronic walkway, CIR systems, Inc) and the Activities-specific Balance Confidence (ABC) questionnaire [20]. These three measures have been found to have moderate to high reliability and validity in samples of older people [19–22]. The FSST is a test of stepping and changing direction which has been found to discriminate between non-fallers, occasional fallers and frequent fallers [19]. The ABC questionnaire is a self-rating scale used to assess balance confidence in performing a range of everyday tasks. It has been well documented that not only is balance confidence related to mobility functioning [20, 23], but decreased confidence may be related to diminished activity due to a fear of falls [24, 25]. Higher scores have been found to correlate with better mobility and lower scores with less mobility [20]. The GAITRite electronic walkway is a portable device capable of measuring many gait parameters [21]. Gait speed was selected as a primary outcome measure as a slower gait speed in older adults has been found to correlate with increased risk of falls and poorer balance [26]. Exploratory analysis of the gait data was also undertaken to investigate which gait parameters were affected by the classes.

The purpose of this study was to investigate whether community dwelling older adults undertaking a series of Feldenkrais Method balance classes improved on measures of mobility and balance. This was a pragmatic study which compared a group of older adults already enrolled in Feldenkrais Method balance classes, with a similar group who received no intervention. Both groups were tested and re-tested on balance and mobility measures at a 3-month time interval, and the changes within and between the groups were compared.

## 2. Methods

### 2.1. Ethics

The project was approved by the Human Research Ethics Committees at the University of Melbourne and the Caulfield General Medical Centre.

### 2.2. Participants

The Intervention group was a sample of convenience drawn from community dwelling older adults, who had enrolled to attend a series of Feldenkrais Method balance classes [27], in a community health setting, as part of a falls prevention program. The Control group was recruited from community dwelling older adults who volunteered in response to an advertisement for participants in a balance study.

Inclusion criteria included being aged over 65, able to walk independently in the community (with or without a gait aid), able to perform the balance tests without a walking frame (a walking stick was permissible) and able to participate in a series of balance classes. All participants provided informed consent. Those currently receiving any additional intervention related to mobility were excluded from the study.

### 2.3. Procedures

The Intervention group participants were assessed on balance and mobility measures prior to starting the classes and at completion of the program. The Control group were tested and retested on the same measures, at an interval of three months, with no intervention.

Testing was performed by one of the investigators and a research assistant trained in the use of the GAITRite instrumented walkway. Testers were not blinded to group allocation, but were blinded to previous results on retesting. Three trials of the FSST were conducted and the fastest speed of the final two trials was used for analysis (as recommended in the protocol described by the developers of the test [19]). Participants performed three trials walking on the GAITRite walkway, and an average speed from the three trials calculated. On each trial, they were instructed to walk at a comfortable pace. Exploratory analysis of gait data was performed, including stride length, cadence (steps/minute), double support time (percentage of the gait cycle when both feet were in contact with the ground) and variability of step length (calculated by dividing the variability on each step length by the mean step length for each participant, to arrive at a coefficient of variability). The ABC score was calculated by adding the score for each question on the questionnaire and dividing by the number of questions, as per protocol [20].

After the completion of the classes, participants in the Intervention group were asked: “Do you think the classes had any effects on you? If yes, what were they?” These questions were asked at the re-testing session by the researcher.

### 2.4. Intervention

A series of Feldenkrais Method balance classes, “Getting Grounded Gracefully” [28] was delivered to the Intervention group by the Feldenkrais Practitioner who devised the program. Classes were conducted for 1 h, twice weekly for 10 weeks. All classes were conducted in sitting, standing or moving within the room. Each of the 20 classes engaged the participants in different movement tasks, such as sit to stand or weight shift in standing. Several postural control themes were continued through the classes. These themes included: control of the pelvis over the base of support in many variations, flexibility and movement control in the ankles and the trunk, enhancing body awareness (such as awareness of the contact of the feet on the floor and paying attention to which parts of the body were engaged in particular movement tasks) and building balance confidence.

### 2.5. Data Analysis

Descriptive statistics were calculated for all outcome measures. The groups were compared at baseline to determine if there were any significant differences between the groups. Parametric tests were used for gait speed, while non-parametric tests were used for FSST, ABC and age as these data were not normally distributed [29].

To evaluate the effect of the intervention for the normally distributed variables (gait speed), an ANCOVA was used to compare post intervention scores, with baseline gait speed as the covariate. This approach has been recommended by Vickers [30] for non-normally distributed data (FSST and ABC), change scores were calculated for each subject, and Mann-Whitney *U*-tests compared change scores between the groups. The mean treatment effect (and 95% CI) of the classes were calculated for each variable. Within group changes, between initial and re-testing, were analyzed using repeated measures statistical tests. All tests were two-tailed tests.

Exploratory analysis of the gait variables: The effect of the intervention was evaluated using an ANCOVA to compare post intervention scores, with baseline scores as the covariate for normally distributed data (cadence, double support and stride variability) and Mann Whitney *U* tests for data not normally distributed (stride length). There was also an investigation of relationships between the variables using Spearman correlation tests as the testing involved data that were not normally distributed. SPSS Graduate Pack v.15.0 was used for all statistical analysis.

## 3. Results

**Figure 1:**
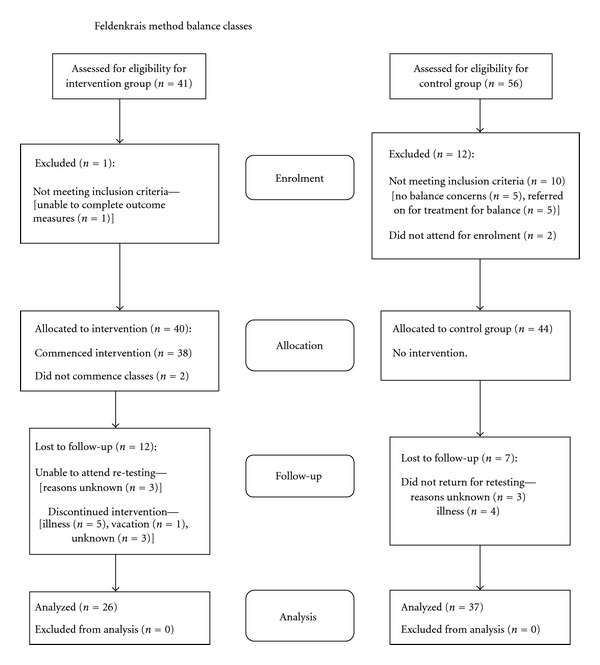
Flow chart of participant recruitment and retention.

Figure 1 shows the flow chart for recruitment and attrition. There were no significant differences on baseline measures between those who dropped out of the study and those who presented for re-testing. Two participants in the Control group were not re-tested on the GAITRite, so there were 35 subjects in this group with data on gait speed for analysis.

### 3.1. Baseline Comparisons

There was no significant difference in age between the Intervention group [median = 75.0 (IQR = 8.0) years] and the Control group [median = 76.5 (IQR = 10.0) years] (*P* = .39). At baseline, all participants were asked about their current health status.  Table 1 displays the co-morbidities reported by both groups. The Control group reported an average of 1.3 (48/37) conditions per person, while the Intervention group reported 1.6 (42/26), indicating similar levels of health status.

Table 2 displays the baseline scores for both groups on the three main outcome measures. Despite being similar in age, the Control group displayed a non-significant trend towards being more mobile than the Intervention group, both on the FSST (*P* = .20) and on gait speed (*P* = .17). The Control group displayed significantly higher scores on the ABC questionnaire (*P* = .014).

### 3.2. ABC Score

**Figure 2:**
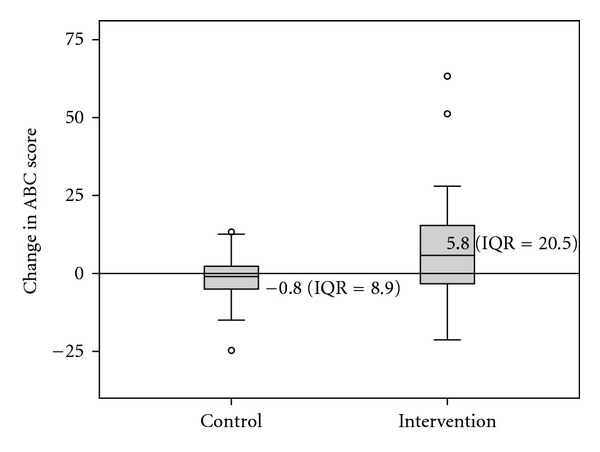
Box plot displaying ABC change scores between initial and retesting for both groups.

Results of the initial and post-tests are provided in Table 2. Non-parametric tests were used as data were not normally distributed. Change scores were found to be significantly different between the Intervention and Control groups (*Z* = −2.80, *P* = .005), as illustrated in Figure 2. The Intervention group was found to have significantly improved between initial and re-testing (*Z* = 2.41, *P* = .016). The Control group had a small though non-significant deterioration in score over this period (*Z* = 1.01, *P* = .31).

### 3.3. Gait Speed

**Figure 3:**
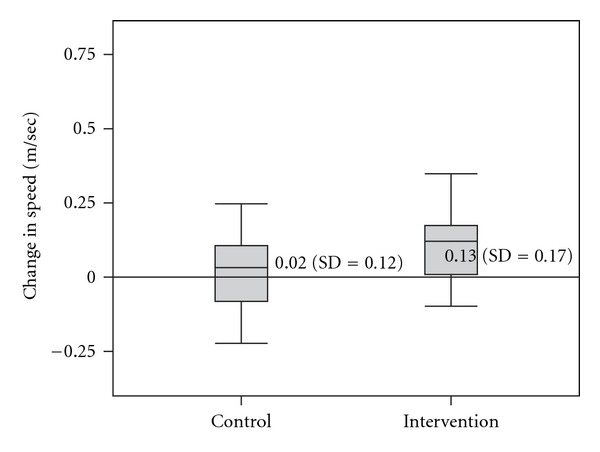
Box plot displaying gait speed change scores between initial and retesting for both groups.

Results of the initial and post tests are provided in Table 2. Change scores were found to differ significantly between groups (*F* = 5.98, *P* = .017), using ANCOVA to test for the main effect of group. Using paired-samples *t*-tests, these changes were found to be significant within the Intervention group (df = 25, *t* = 3.75, *P* = .001), but not within the Control group (df = 36, *t* = 1.01, *P* = .32) as illustrated in Figure 3.

### 3.4. FSST

**Figure 4:**
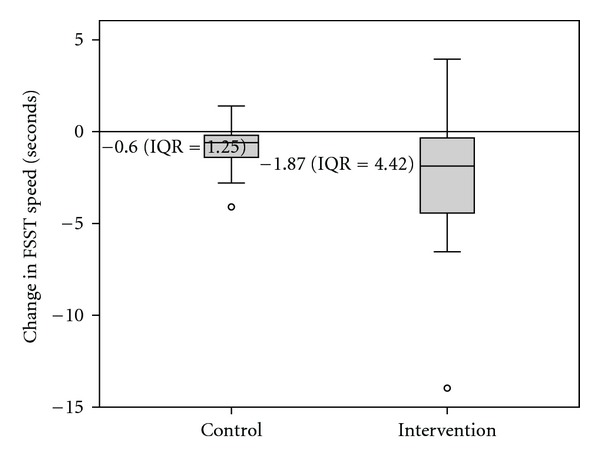
Box plot displaying FSST change scores between initial and retesting for both groups.

Results of the initial and post-tests are provided in Table 2. Change scores were significantly different between the Intervention and Control groups (*Z* = −2.28, *P* = .022) as illustrated in Figure 4. Wilcoxon Signed Ranks tests showed that both the Intervention group (*Z* = 3.43, *P* = .001) and the Control group (*Z* = 3.9, *P* < .001) improved significantly on this measure.

### 3.5. Exploratory Analysis of Gait Data

Analysis of several gait variables, comparing changes both within groups and between groups is presented in Table 3. Compared to the Control group, the Intervention group significantly increased their cadence by 5.02 steps/min (95% CI 1.49–8.62, *F* = 9.59, *P* = .003). The intervention group made significantly more improvement in stride length than the Control group (*Z* = −2.17, *P* = .03). Neither double support time (*F* = 0.09, *P* = .76) nor stride variability (*F* = 0.023, *P* = .88) change scores were found to differ significantly between groups, using ANCOVA to test for the main effect of group.

### 3.6. Participant Comments

The Intervention group participants made comments on several aspects of balance and mobility that had been affected by the classes. Twenty-one of the participants had noticed changes which they felt were related to the classes, and five said they had noticed no changes. Eight people commented on improvements in walking. Seven commented on feeling more confident. Thirteen commented on changes to body image, such as “Makes you think about the soles of the feet on the ground”. Ten mentioned improvement in functional activities, including walking on slopes and taking the dog for a walk.

## 4. Discussion

Results of this study showed that participants attending the Feldenkrais Method classes made statistically significant improvements on a number of balance measures compared to a non-intervention Control group. The Feldenkrias Method may therefore be a useful approach to improving balance in older adults. The group attending the classes made improvements in both psychological and physical domains of balance measurement. 

### 4.1. Balance Confidence

The improvement in scores on the self-rated ABC questionnaire suggested that the Intervention group felt more confident in their balance while performing a variety of tasks. This increased confidence in undertaking everyday activities was substantiated by the participants' comments about the effects of the classes. These comments suggested there had been a translation from skills learnt in the classes to improvement in everyday functional activities.

The median ABC score, for the Intervention group in the current study, increased from 68.7 to 81.7 (18.9%). These results compare well to Sattin and Wolf's study of Tai Chi to improve balance [7], which found an increase of five points on the ABC, over a 4-month period, or Liu-Ambrose's study of Tai Chi and balance [31] which recorded a 6% improvement in ABC score (from a mean of 78.3 points to 83.2 points).

The difference in the ABC scores between groups at baseline may have affected the results, as perhaps the Intervention group, who scored lower at initial testing, were more likely to score higher on retesting due to a regression to the mean. To investigate this possibility, the authors examined the results of a subgroup of the Control group who scored a median of 74.7 (IQR = 18.3) on the ABC. This score was not significantly different from the Intervention group median score at baseline of 68.7 (IQR = 18.2). This lower-scoring subgroup of the Control group, who were similar to the Intervention group in initial scores, made a slight decrease in score over time [−1.3 (IQR = 16.3)], unlike the Intervention group who improved over time. This provides some support to the contention that the improvement observed in the Intervention group was probably not a regression to the mean.

### 4.2. Gait Speed and Other Gait Parameters

For gait speed, the mean treatment effect of 0.11 m s^−1^ represented a 9.7% increase in speed attributable to the classes. Wayne's 2004 [9] review of 30 Tai Chi studies included one study which measured gait speed, and Gardner's 2000 [32] review of exercise as a balance intervention included two studies, neither of which found a significant change in gait speed. The 9.7% increased speed in the Intervention group in the current study compares favorably to a 6% increase in gait speed observed in people who had participated in Tai Chi sessions [8].

The increased gait speed was achieved through both longer step lengths and increased cadence, with an associated decrease in double support time. The faster gait speed in the Intervention group may have been due to increased confidence [23]. Fear of falling has been shown to alter postural control to produce “stiffer” movement patterns [33], so decreased fear may have enabled a “freer” gait style, with longer steps and increased speed. The faster speed may also have resulted from improved intersegmental control between the lower limbs, pelvis, trunk and head.

### 4.3. Dynamic Balance

Both groups made significant improvements between initial and retesting sessions on the FSST. The improvement by almost all participants suggests that there may be a learning effect on the task, and that caution should be exercised if it is used as an outcome measure for clinical trials. Despite both groups improving significantly, the Intervention group still made significantly more improvement than the Control group on this measure, suggesting that their ability to step in all directions and change direction in space had improved.

### 4.4. A Novel Approach to Balance Training

The Feldenkrais Method differs from other exercise approaches in several ways. Firstly it is an exploratory learning approach based on dynamics systems principles [34]. Participants are allowed to progress at their own pace, gradually expanding their “perceptual-motor workspace” or “movement envelope” as described by Karl Newell [35]. These ideas about dynamic systems and human movement control have been recently discussed by Bardy et al. [36], in relation to the “self-organizing” capacity of biological systems such as the human. He states that “behavior emerges from the interaction of multiple sub-systems, including experience” (page 500). The relevance of this thinking to the current study is that participants were not taught specific strategies to improve their balance, but were presented with many opportunities for learning and allowed to work out solutions for themselves. There was no “right" way to do each movement, but instead each repetition was viewed as an exploration. Participants gained confidence in exploring the space around themselves in their own way and time, resulting in expanded perceived limits of stability as they practice moving their centre of mass close to the edge of the base of support in many directions. This approach allows older people the time to gradually build their movement skills and repertoire of solutions to movement challenges.

Another difference between the Feldenkrais Method balance classes and other approaches to balance retraining is the variability of the training. It has been stated that “… when practice is varied by changing aspects of the environmental context or the task, the motor skill that develops is more flexible and generative in type” [37] (page 96). Feldenkrais Method balance classes have greater variety and variability than standard balance training programs such as that described by Gardner [3], which consisted of about 12 balance exercises repeated over many sessions (with grading for increasing the difficulty of most of these exercises). In Tai Chi balance classes certain forms of movement are practiced over and over again [7, 8]. The Feldenkrais Method balance classes consisted of a series of individual lessons, each one different. Within each lesson, the movement tasks were systematically varied after about twenty repetitions of each action, including variations to direction, speed, amplitude and intersegmental timing of the action. For example, rotation was practiced first with the eyes leading the movement, then the shoulders leading, then the pelvis, then the knees, then different combinations of the above body parts. This variability of practice has been considered an important principle to be included in motor skill acquisition training [35].

Finally, the Feldenkrais Method, influenced by its martial arts origins, seeks to engage every part of the person in the movements, from the toes, to the trunk, to the eyes and the breath. The movement classes also have an emphasis on improving movement control of the pelvis, to improve both power in movement and the control of the centre of gravity. This concept is again related to martial arts principles [11], and translates well into training to improve balance in everyday function. Indeed the “practice of controlled movements of the centre of mass” has been identified as one of the most important components of a balance training program for older adults to prevent falls [10] (page 2234).

### 4.5. Study Limitations

One limitation of this study was the lack of randomization between groups, due to the pragmatic nature of this pilot study. The Intervention group was a sample of convenience, recruited from people already enrolled into a series of Feldenkrais Method balance classes. Although the researchers attempted to recruit a similar group to act as a control, the Control group was more confident in their balance than the Intervention group. This limitation led to the baseline differences between the groups, which has already been discussed.

The lack of blinding of the testers to the group allocation of the subjects was a potential source of bias. This was countered by the assessors giving exactly the same instructions to all participants on all occasions, and the assessors were blinded to baseline results at re-test. As with many interventions in the rehabilitation setting, it was impossible to blind subjects to the intervention in this type of clinical trial.

There were no adverse effects such as falls or reports of injuries during the classes.

## 5. Conclusion

Participants in Feldenkrais Method balance classes improved in several measures of balance and mobility compared a Control group who received no intervention. It appears that the Feldenkrais Method, which uses an exploratory learning approach based on an understanding of dynamic systems, may add some useful dimensions to the retraining of balance.

## Figures and Tables

**Table 1:** Health status of participants at baseline.

Control group (*n* = 37)	Intervention group (*n* = 26)
Arthritis/musculoskeletal	Arthritis/musculoskeletal
(conditions = 17)	(conditions = 16):
Back problems: 7	Arthritis: 10
Arthritic knees: 4	Joint replacements: 3
Joint replacements: 3	Recent fractures (past few years): 3
Heel spur: 1 Recent fractures (past few years): 1	
Polymyalgia rheumatica: 1	
Medical (conditions = 20):	Medical (conditions = 21):
Cardiac: 7	Cardiac: 2
Cancer: 2	Cancer: 1
Hypotension: 3	Hypertension: 6
Hypertension: 4	Other: Diabetes: 4, renal problems: 1,
Other: Diabetic: 1, COPD: 1,	coeliac disease: 1, increased bone density: 1
edematous legs: 1, renal problems: 1	(Piaget's disease), osteoporosis: 1,
(on dialysis 3/week)	gout: 1, ulcer on ankle: 1, fluid on lungs: 1, asthma: 1
Neurological (conditions = 11):	Neurological (conditions = 5):
Stroke: 4	Stroke: 2
Left foot drop: 1 (peripheral neuropathy)	Polio: 1 (66 years ago)
“Fluid on the brain”: 1 (shunt in situ)	Ménière's disease: 1
Brain tumor: 1 (ongoing medication)	Transient Ischemic Attacks: 1
Long term anti-epileptic medication: 1	
Spinal cord injury due to spinal cancer: 1	
(weakness and sensory changes in legs)	
Spinal canal stenosis: 1 (resulting in right leg weakness)	
Parkinson's disease: 1	
Mobility:	Mobility:
Gait aids: stick 4, frame 1	Gait aids: stick 3, frame (outdoors) 2

**Table 2:** Comparison between groups at baseline, re-testing and difference between groups (95% CI) for Intervention group (*n* = 26) and Control group (*n* = 37).

Outcome	Groups				Difference within groups (change scores)		Mean treatment effect
	Baseline		Re-testing		Re-testing minus baseline		Difference between groups
	Intervention	Control	Intervention	Control	Intervention	Control	Intervention minus control
ABC score median (IQR)	68.70 (18.2)	81.30^a^ (21.7)	81.85 (14.9)	83.00 (24.1)	5.80 (20.5)^b^	−0.80 (8.88)	11.31 (19.2 − 3.43)^a^
FSST (s) median (IQR)	12.3 (4.6)	11.4 (3.7)	9.96 (3.3)	9.95 (3.8)	−1.87 (4.42)^b^	−0.60 (1.25)^b^	1.5 (0.23 − 2.76)^a^
Gait speed (m s^−1^) mean (SD)	1.01 (0.25)	1.10 (0.28)	1.14 (0.2)	1.13 (0.26)	0.13 (0.17)^b^	0.02 (0.12)	0.11 (0.18–0.03)^a^

^a^A significant difference found between Control and Intervention groups.

^b^A significant difference found within a group between baseline and re-testing.

**Table 3:** Exploratory gait variables.

Gait variable	Intervention group			Control group		
	Baseline	Re-test	Change score	Baseline	Re-test	Change score
Stride length (cm)	110.4 (24.3)	117.56 (18.4)^a^	7.15 (13.1)	120.8 (22.1)	121.3 (19.6)	0.50 (8.5)^b^
Cadence (steps/min)	110.16 (11.2)	116.30 (10.0)^a^	6.14 (7.7)	109.49 (11.6)	110.57 (11.73)	1.08 (6.2)^b^
Double support time (percentage of cycle)	24.18 (4.7)	23.33 (3.7)	−0.85 (2.8)	24.08 (3.4)	23.41 (3.5)^a^	−0.67 (1.6)
Variability of step length (coefficient of variability)	5.10 (2.2)	4.50 (2.4)	0.00 (2.4)	4.92 (3.8)	4.08 (3.2)	−0.32 (2.7)

All values are means (SDs), except those describing stride length which are medians (inter-quartile ranges).

^a^A significant difference found within a group between baseline and re-testing.

^b^A significant difference found between Control and Intervention groups.
